# Individual Daytime Noise Exposure during Routine Activities and Heart Rate Variability in Adults: A Repeated Measures Study

**DOI:** 10.1289/ehp.1205606

**Published:** 2013-03-19

**Authors:** Ute Kraus, Alexandra Schneider, Susanne Breitner, Regina Hampel, Regina Rückerl, Mike Pitz, Uta Geruschkat, Petra Belcredi, Katja Radon, Annette Peters

**Affiliations:** 1 Institute of Epidemiology II, Helmholtz Zentrum München, Neuherberg, Germany; 2 Institute for Medical Informatics, Biometrics and Epidemiology, Ludwig-Maximilians-Universität (LMU) München, Munich, Germany; 3 ESC-Environment Science Center, University of Augsburg, Augsburg, Germany; 4 Unit for Occupational and Environmental Epidemiology and NetTeaching, Institute and Outpatient Clinic for Occupational, Social and Environmental Medicine of the University Hospital in Munich (LMU), Munich, Germany; 5 Munich Heart Alliance, Munich, Germany

**Keywords:** autonomic nervous system, epidemiology, heart rate variability, noise exposure, short-term changes

## Abstract

Background: Epidemiological studies have demonstrated associations between noise exposure and cardiovascular events. However, there have been few studies of possible underlying mechanisms.

Objectives: We examined the association between individual daytime noise exposure and heart rate variability (HRV).

Methods: In a prospective panel study in Augsburg, Germany (March 2007–December 2008), 110 individuals participated in 326 electrocardiogram recordings with a mean duration of 6 hr. Five-minute averages of heart rate (HR) and HRV parameters were determined. Individual noise exposure was measured as A-weighted equivalent continuous sound pressure levels (*L*_eq_). Effects were estimated using additive mixed models adjusted for long- and short-term time trends and physical activity. Due to nonlinear exposure–response functions, we performed piecewise linear analyses with a cut-off point at 65 dB(A).

Results: Concurrent increases of 5dB(A) in *L*_eq_ < 65dB(A) were associated with increases in HR (percent change of mean value: 1.48%; 95% CI: 1.37, 1.60%) and the ratio of low-frequency (LF) to high-frequency (HF) power (4.89%; 95% CI: 3.48, 6.32%), and with decreases in LF (–3.77%; 95% CI: –5.49, –2.02%) and HF (–8.56%; 95% CI: –10.31, –6.78%) power. Standard deviation of normal-to-normal intervals (SDNN) was positively associated with concurrent noise < 65dB(A) (5.74%; 95% CI: 5.13, 6.36) but negatively associated with noise lagged by 5–15 min (–0.53% to –0.69%). Associations with cardiac function were less pronounced for noise ≥ 65dB(A), with some in opposite directions from associations with noise < 65dB(A). Concurrent associations were modified by sex and age.

Conclusions: Individual daytime noise exposure was associated with immediate changes in HRV, suggesting a possible mechanism linking noise to cardiovascular risk. Noise at lower levels may have health consequences beyond those resulting from “fight-or-flight” responses to high levels of noise.

Epidemiological studies indicate that noise exposure is associated with adverse cardiovascular health effects (Babisch 2006; Ising and Kruppa 2004; Tomei et al. 2010). More precisely, studies on chronic noise exposure have suggested an association with elevated blood pressure (Chang et al. 2003; Fogari et al. 2001), hypertension or the use of antihypertensive medication (Barregard et al. 2009; Bluhm et al. 2007; de Kluizenaar et al. 2007; Jarup et al. 2008), ischemic heart disease including myocardial infarction (MI) (Babisch et al. 2005; Selander et al. 2009), and mortality from MI (Huss et al. 2010). Studies of short-term cardiovascular effects have reported elevated blood pressure levels and increased heart rate (HR) in association with noise exposure (Chang et al. 2009; Haralabidis et al. 2008; Lusk et al. 2004). Most previous studies have focused on effects of higher noise intensities that were generated by specific sources, particularly aircraft, road traffic, and occupational noise and noise produced in laboratory settings. Information about effects of individual noise exposure during everyday life, which may include a wide range of noise intensities, is very limited.

Underlying mechanisms linking noise to enhanced cardiovascular risk are rarely explored in epidemiological studies. A potential mechanistic pathway is that noise exposure serves as a stressor that increases the sympathetic tone of the autonomic nervous system, either directly or indirectly via hormone release, resulting in a “fight-or-flight” reaction (Babisch 2003; Babisch et al. 2001; Henry 1992; Ising et al. 2003). An effect of noise on the autonomic nervous system may be assessed through time- and frequency-domain analysis of heart rate variability (HRV) (Malik 1996). Decreased HRV is considered a risk factor for adverse cardiovascular events (Buccelletti et al. 2009; Gerritsen et al. 2001). For instance, a reduction in the standard deviation of normal-to-normal intervals (SDNN) is a better predictor of death due to progressive heart failure than are other conventional clinical measurements (Nolan et al. 1998). However, there have been relatively few studies of the association between noise exposure and HRV, and results have been inconsistent. Two experimental studies that examined the effects of white noise, which contains every frequency within the range of human hearing in equal amounts, found increased low frequency (LF) power but no changes in high frequency (HF) power in association with short-duration white noise, consistent with an effect mediated by an increase in sympathetic tone (Björ et al. 2007; Lee et al. 2010). In contrast, authors of a recent field study reported a decrease in respiratory sinus arrhythmia associated with indoor traffic noise exposure during sleep, consistent with an effect mediated by a reduction in parasympathetic tone (Graham et al. 2009).

The objective of the present epidemiological study was to provide further insight into the biological mechanism of cardiovascular health effects associated with noise by investigating the acute effects of routine daytime noise exposure on HR and HRV parameters in individuals.

## Methods

*Study design*. As part of the Rochester Particulate Matter Center investigations, a prospective panel study was conducted in Augsburg, Germany, between 19 March 2007 and 17 December 2008. Participants were recruited from the follow up examination of the KORA (Cooperative Health Research in the Region of Augsburg) survey 2000 (Holle et al. 2005), which was conducted in 2006–2008. In a baseline interview, participants gave information on health status, medication use, disease status, and smoking history. Because of several other objectives of the study, general exclusion criteria were smoking during the preceding 12 months, intake of platelet aggregation inhibitors except for acetylsalicylic acid, an MI and/or interventional procedure (e.g., bypass surgery) < 6 months before study entry, and chronic inflammatory diseases such as Crohn’s disease, colitis ulcerosa, or rheumatoid arthritis. In addition, participants were excluded from the present analysis if they had an implanted pacemaker, atrial fibrillation, allergy to latex, or thrombosis or a shunt in an arm.

Participants were invited to complete up to four repeated electrocardiogram (ECG) recordings and individual exposure measurements. The examinations were scheduled every 4–6 weeks on the same weekday between 0730 and 1500 hours. During the measurement periods, participants were free to pursue their daily routines. Participants recorded all of their activities and whereabouts in a diary, and were asked to note whenever they felt annoyed by noise. For detailed information on the diary, see Supplemental Material, p. 2 (http://dx.doi.org/10.1289/ehp.1205606). A variable indicating physical activity was derived by quantifying each diary entry on the basis of a metabolic equivalent unit (Peters et al. 2005). The categories were *1*) sleeping, *2*) reclining, *3*) very light to light exertion (e.g., eating, reading, cooking, slow walking, car driving), *4*) moderate exertion, with deep breathing (e.g., biking, light gardening, vacuum cleaning), *5*) vigorous exertion, with panting (e.g., jogging, heavy gardening, climbing stairs), and *6*) heavy exertion, with gasping (running, shoveling heavy snow).

Written informed consent was obtained from all participants. The study protocol was approved by the German Ethics Committee of the Bayerische Landesärztekammer, Munich, Germany.

*ECG monitoring and HRV parameters*. To assess cardiac rhythm, participants were equipped with a 12-lead Mortara H12 digital Holter recorder (Mortara Instrument, Milwaukee, WI, USA). ECG recordings were analyzed at the University of Rochester Medical Center (Rochester, NY, USA), and ECG parameters were computed according to Task Force of the European Society of Cardiology and the North American Society of Pacing and Electrophysiology recommendations (Malik 1996). In addition to HR we evaluated the time-domain HRV parameters SDNN and RMSSD (root-mean square of successive differences), and the frequency-domain HRV parameters LF power (0.04–0.15 Hz, normalized units), HF power (0.15–0.40 Hz, normalized units), and the LF:HF ratio. Five-minute averages of HR and time-domain HRV parameters were determined for every 5-min interval with at least 200 beats recorded, and 5-min averages of frequency-domain parameters were determined for intervals with at least 300 beats recorded. Only individuals with at least one ECG recording with a duration of > 2 hr were included in analyses.

*Individual exposure*. Measurements were made using a noise dosimeter (Spark® model 703; Larson Davis Inc., Depew, NY, USA) with the microphone attached to the participant’s collar close to the ear. These instruments were successfully applied in a previous study (Weinmann et al. 2012). Noise exposure was measured as A-weighted equivalent continuous sound pressure levels (*L*_eq_) reported in units of A-weighted decibels [dB(A)]. The A-weighted system is an expression of the relative loudness of sounds as perceived by the human ear. The dosimeters were calibrated once a week and had a measurement range of 40–115 dB with a detector accuracy of < 0.7 dB error. Measurements below the lower limit of detection (LOD) were assigned a value of 37 dB, and those above the upper LOD were assigned a value of 115 dB (Radon 2007). In addition to noise, particle number concentrations (PNC)—an indicator for ultrafine particles—were measured using a portable condensation particle counter (model 3007; TSI Inc., Shoreview, MN, USA) that covered a diameter range from 10 nm to 1 µm. For both, *L*_eq_ and PNC, 5-min averages were temporally aligned to the 5-min averages of the outcome data and were determined if at least two-thirds of the values in a 5-min segment were available.

*Statistical analyses*. To assess acute effects of individual noise exposure on ECG parameters, we applied additive mixed models with a random participant effect to adjust for differences in individual levels of cardiac rhythm between all participants. To account for correlations between repeated ECG measures within the same individual, we used a compound symmetry covariance structure and included the lagged outcome in the model. Except for HR, all outcome variables were log-transformed to produce normally distributed residuals. We analyzed each ECG parameter in separate models adjusted for a set of confounders that minimized Akaike’s information criterion (Akaike 1973). Long-term and daily time trend were forced into all models, along with physical activity. Trend variables were modeled as untransformed linear variables or, using penalized splines or polynomials (up to 4 degrees) to allow for nonlinear exposure–response functions, to optimize model fit (Greven et al. 2006). Weekday and season were evaluated as potential confounders but were not included in final models because they did not improve model fit. Additionally, all HRV parameter models were adjusted for HR. In addition to including concurrent *L*_eq_ in the final models, all models included variables indicating *L*_eq_ lagged in 5-min intervals up to 15 min (0–5, 5–10, and 10–15 min). Covariates included in the final models for each outcome are listed in Supplemental Material, Table S1 (http://dx.doi.org/10.1289/ehp.1205606).

To assess the potential for overcontrolling by HR, we also evaluated associations between HRV parameters and *L*_eq_ without adjusting for HR. Results were consistent for all parameters except RMSSD, which showed associations with *L*_eq_ that were in opposite directions depending on adjustment (data not shown). Therefore, we considered the association to be unstable and do not report results for RMSSD here.

A preliminary analysis showed nonlinear exposure–response functions for associations between concurrent noise and all ECG parameters [see Supplemental Material, Figure S1 (http://dx.doi.org/10.1289/ehp.1205606)]. Therefore, we modeled noise exposure as a piecewise linear term with a cut-off point at 65 dB(A), and present separate estimates for associations with a 5-dB(A) increase in *L*_eq_ for *L*_eq_ < 65 dB(A) and *L*_eq_ ≥ 65 dB(A). Furthermore, we assessed whether associations were modified by sex or age (< 65 years vs. ≥ 65 years) by performing stratified analyses. For sensitivity analyses, we excluded all participants with hearing impairment and with intake of beta-adrenergic receptor blockers (beta-blockers). Moreover, we additionally adjusted our models for the diary-based information on the whereabouts of the participants as a proxy indicator of the noise source. In a further analysis, we included PNC exposures with the same lags as *L*_eq_ in the models to examine potential confounding by ultrafine particle exposures. Effect estimates are presented as percent changes in the mean values of each outcome together with 95% CIs. Data were analyzed with SAS statistical package (version 9.2; SAS Institute Inc., Cary, NC, USA).

## Results

*Study population*. Overall, 110 individuals participated in 385 visits including ECG and individual exposure measurements. Baseline characteristics of the 110 individuals are described in Table 1. Fifty-nine measurements were not valid because of missing data due to technical problems or bad signal quality of the ECG recordings. Thus, 326 valid measurements with a mean duration of 6 hr were available for analyses, comprising approximately 20,000 5-min segments. Women were on average younger than men, but disease status and medication use was comparable between women and men [see Supplemental Material, Table S2 (http://dx.doi.org/10.1289/ehp.1205606)]. Persons < 65 years of age were less likely to report a metabolic disorder or hypertension, reported less medication use, and were more likely to be employed than persons ≥ 65 years (see Supplemental Material, Table S3).

**Table 1:** Baseline characteristics of the study population (*n* = 110).

Variable	n (mean ± SD or %)
Age (years)	110 (61.3 ± 11.7)
Body mass index (kg/m2)	110 (28.6 ± 5.3)
Male	69 (62.7)
Smoking history
Never smoker	59 (53.6)
Ex-smoker	51 (46.4)
Metabolic disorder (T2D, IGT)a	64 (58.2)
Self-reported historyb
MI	6 (5.5)
Angina pectoris	6 (5.5)
Coronary heart disease	7 (6.4)
Hypertension	61 (55.5)
Use of medicationc
Agents acting on renin-angiotensin system	40 (36.4)
Beta blocker	28 (25.5)
Calcium channel blockers	11 (10.0)
Antidiabetics	18 (16.4)
Diuretics	36 (32.7)
Nitrates	1 (0.9)
Statins	19 (17.3)
Antihypertensive drugs	54 (49.1)
Hearing impairmentd	15 (13.6)
If yes:
Physician diagnosed	12 (10.9)
Wearing hearing aid	2 (1.8)
Employed (%)	41 (37.3)
Abbreviations: IGT, impaired glucose tolerance; T2D, type 2 diabetes. aParticipants with T2D were classified based on a self-reported diagnosis by a physician, medication use, or a fasting glucose level > 125 mg/dL or a 2-hr glucose level ≥ 200 mg/dL in an oral glucose tolerance test (OGTT). IGT was classified based on 2-hr OGTT glucose levels ≥ 140 mg/dL but < 200 mg/dL. bEver physician diagnosed. cAt least once during the study period (19 March 2007 to 17 December 2008). dNot validated.	

*Diary*. Overall, the participants made 4,165 diary entries with on average 12.8 entries per visit. However, only 4,148 diary entries were included in the analyses because for 17 entries physical activity could not be assigned clearly to one category. Participants spent more than half of the time indoors and showed very low variation in physical activity [see Supplemental Material, Table S4 (http://dx.doi.org/10.1289/ehp.1205606)]. More than 90% of the time physical activity was classified as very light or light. Because of the small numbers in the lowest and highest categories, we combined categories 1 and 2 as well as 4, 5, and 6, respectively. Twenty-six participants reported 43 episodes of annoyance by noise over a total of 34 visits. However, we did not evaluate annoyance further because data on the time, duration, and intensity of annoyance were often incomplete.

*ECG parameters and exposure*. Descriptive statistics of noise, PNC, and ECG parameters are shown in Table 2. Mean level of personal noise exposure [75.1 dB(A)] was quite high. However, as expected, there existed very much variation from this average [SD = 83.0 dB(A)] resulting from combining such a huge number of observations collected in several different situations. HF power and the LF:HF ratio showed the highest correlation of the outcomes (*r* = –0.59), with weaker correlations (–0.02 to 0.41) between other pairs of ECG parameters [see Supplemental Material, Table S5 (http://dx.doi.org/10.1289/ehp.1205606)]. The correlation between *L*_eq_ and PNC was *r* = 0.15. Women and men were on average exposed to similar noise levels [75.8 dB(A) in women vs. 74.6 dB(A) in men, *p* = 0.34]. Women had higher values of HR and HF power than men, but no differences were seen for the other ECG parameters (data not shown). Compared to the older age group, individuals < 65 years were exposed to higher levels of *L*_eq_ [76.9 dB(A) vs. 72.0 dB(A), *p* = 0.01] and had higher ECG parameter values except for HF power (data not shown).

**Table 2:** Descriptive statistics of 5-min averages of Leq, PNC, and ECG measures.

Variable	All		Leq < 65 dB(A)		Leq ≥ 65 dB(A)		p-Valuea
	n	(Mean ± SD)	n	(Mean ± SD)	n	(Mean ± SD)	
Leq [db(A)]	21,419	(75.1 ± 83.0)	8,818	(60.4 ± 59.7)	12,601	(77.3 ± 84.1)	< 0.0001
PNC (n)	17,368	(21,236 ± 34,039)	7,423	(17,358 ± 28,054)	9,945	(24,131 ± 37,638)	< 0.0001
HR (beats/min)	21,419	(78.4 ± 14.7)	8,818	(75.1 ± 14.1)	12,601	(80.8 ± 14.7)	< 0.0001
SDNN (msec)	21,415	(51.6 ± 26.9)	8,816	(51.0 ± 27.4)	12,599	(52.0 ± 26.4)	< 0.0001
LF power (nu)	18,722	(44.4 ± 28.0)	7,331	(45.8 ± 27.1)	11,391	(43.4 ± 28.5)	< 0.0001
HF power (nu)	18,722	(15.3 ± 15.2)	7,331	(16.6 ± 15.8)	11,391	(14.3 ± 14.6)	< 0.0001
LF:HF ratio (nu)	18,722	(5.3 ± 4.9)	7,331	(5.1 ± 4.9)	11,391	(5.4 ± 4.9)	0.0002
nu, normalized units. ap-Value of fixed effect for noise indicator in an univariate mixed model to test the differences in associations according to Leq < 65dB(A) and Leq ≥ 65dB(A).							

**Figure 1:**
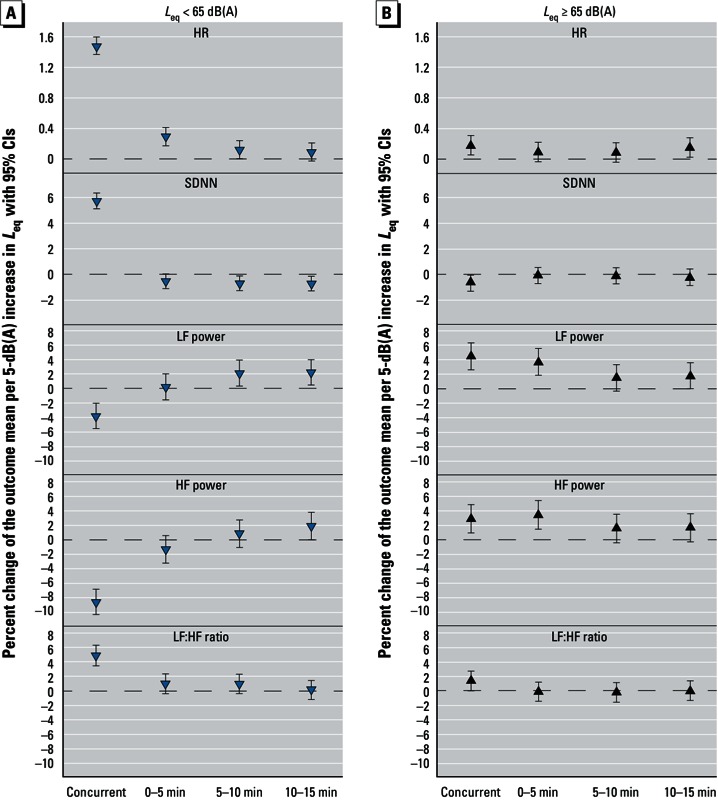
Adjusted associations between ECG measures and a 5‑dB(A) increase in 5-min averages of noise exposure < 65 dB(A) (*A*) and ≥ 65 dB(A) (*B*). See Supplemental Material, Table S6 (http://dx.doi.org/10.1289/ehp.1205606) for numeric data.

*Association of noise and ECG parameters.*The estimated percent changes in the mean values of each outcome associated with a 5-dB(A) increase in *L*_eq_ are shown in Figure 1 [for numeric data, see also Supplemental Material, Table S6 (http://dx.doi.org/10.1289/ehp.1205606)]. HR and the LF:HF ratio increased in association with noise exposure above and below 65 dB(A), with stronger associations estimated for concurrent increases in *L*_eq_ < 65 dB(A) (HR: 1.48%; 95% CI: 1.37, 1.60% and 0.18%; 95% CI: 0.05, 0.31%, respectively; LF:HF ratio: 4.89%; 95% CI: 3.48, 6.32 and 1.38%; 95% CI: 0.03, 2.75%, respectively). A 5-dB(A) increase in *L*_eq_ < 65 dB(A) was associated with an immediate increase in SDNN (5.74%; 95% CI: 5.13, 6.36%) followed by decreases for lagged exposures that were significant when lagged 5–10 min (–0.67%; 95% CI: –1.26, –0.12%) and 10–15 min (–0.67%; 95% CI: –1.26, –0.13%). An increase in *L*_eq_ ≥ 65 dB(A) was associated with a small reduction in concurrent SDNN (–0.67%; 95% CI: –1.30, –0.04%), but was not associated with lagged SDNN. LF and HF power decreased with concurrent noise < 65 dB(A) (–3.77%; 95% CI: –5.49, –2.02% and –8.56%; 95% CI: –10.31, –6.78, respectively), but lagged noise was positively associated with LF power (2.14% to 2.24%). In contrast, 5-dB(A) increases in *L*_eq_ ≥ 65 dB(A) were associated with increased LF and HF power that were statistically significant for concurrent noise (4.42%; 95% CI: 2.59, 6.32% and 2.89%; 95% CI: 0.95, 4.87%, respectively) and lagged noise at 0–5 min (3.69%; 95% CI: 1.86, 5.56% and 3.45%; 95% CI: 1.50, 5.44%, respectively).

Stratified analyses focused on immediate effects, because we found strongest associations with concurrent noise in the main analyses. Associations with a 5-dB(A) increase in concurrent noise < 65 dB(A) were stronger in women than men for HR, HF power, and the LF:HF ratio (*p* for interaction *≤* 0.002), but there were no significant differences between men and women for associations with noise ≥ 65 dB(A) (Table 3). Associations with concurrent noise < 65 dB(A) were stronger among those ≥ 65 years of age for SDNN and the LF:HF ratio, whereas associations with increases in *L*_eq_ ≥ 65 dB(A) were stronger in those < 65 years, with significant differences between the age groups for HR, LF power, and the LF:HF ratio (Table 4).

**Table 3:** Adjusted immediate associations between 5-min averages of noise exposure and ECG measures by sex [percent change (95% CI)].

ECG measures	Male	Female	p-Valuea
< 65 dB(A)			
HR	1.37 (1.24, 1.51)	1.74 (1.52, 1.96)	< 0.0001
SDNN	5.44 (4.71, 6.18)	6.36 (5.24, 7.48)	0.091
LF power	–3.40 (–5.49, –1.26)	–3.92 (–6.91, –0.83)	0.39
HF power	–6.56 (–8.73, –4.33)	–12.11 (–15.00, –9.11)	0.0017
LF:HF ratio	2.93 (1.21, 4.69)	8.88 (6.42, 11.40)	< 0.0001
≥ 65 dB(A)			
HR	0.14 (–0.02, 0.30)	0.21 (0.00, 0.42)	0.27
SDNN	–0.80 (–1.61, –0.01)	–0.54 (–1.52, 0.45)	0.34
LF power	3.38 (1.05, 5.77)	6.09 (3.10, 9.16)	0.083
HF power	2.69 (0.20, 5.24)	3.66 (0.59, 6.84)	0.32
LF:HF ratio	0.61 (–1.15, 2.39)	2.13 (0.04, 4.26)	0.14
ap-Value for interaction calculated using a method proposed by Altman and Bland (2003).			

**Table 4:** Adjusted immediate associations between 5-min averages of noise exposure and ECG measures by age group [percent change (95% CI)].

ECG measures	< 65 years	≥ 65 years	p-Valuea
< 65 dB(A)			
HR	1.39 (1.21, 1.57)	1.61 (1.46, 1.75)	0.20
SDNN	4.87 (4.01, 5.74)	5.93 (5.05, 6.81)	0.047
LF power	–4.50 (–6.80, –2.15)	–1.84 (–4.45, 0.85)	0.069
HF power	–8.10 (–10.45, –5.69)	–8.97 (–11.57, –6.29)	0.32
LF:HF ratio	3.91 (2.07, 5.77)	6.99 (4.77, 9.25)	0.019
≥ 65 dB(A)			
HR	0.27 (0.10, 0.44)	–0.02 (–0.21, 0.17)	0.0098
SDNN	–0.82 (–1.58, –0.06)	–0.28 (–1.32, 0.78)	0.20
LF power	6.33 (4.04, 8.68)	0.61 (–2.39, 3.71)	0.0019
HF power	3.53 (1.16, 5.97)	1.63 (–1.63, 5.00)	0.18
LF:HF ratio	2.62 (0.99, 4.26)	–0.82 (–3.13, 1.53)	0.0093
ap-Value for interaction calculated using a method proposed by Altman and Bland (2003).			

*Sensitivity analyses*. Associations were comparable after exclusion of 15 hearing-impaired participants, except for a slightly weaker association between SDNN and concurrent *L*_eq_ < 65 dB(A) overall (5.20%; 95% CI: 4.55, 5.85%) and among men (but not women) in stratified analyses (4.51%; 95% CI: 3.74, 5.29%). Associations were also comparable after we excluded 30 persons (88 valid visits) who reported beta-blocker intake, except for stronger overall associations between increases in *L*_eq_ < 65 dB(A) and concurrent HR and HF power and the LF:HF ratio lagged 0–5 min (HR: 1.60; 95% CI: 1.46, 1.75%; HF power: –2.36; 95% CI: –4.46, –0.22%; LF:HF ratio: 1.82%; 95% CI: 0.311, 3.36%). Adjusting for the whereabouts of the participants (as a proxy indicator of noise source) had little influence on associations, except for weaker associations between HR and increases in *L*_eq_ < 65 dB(A) overall (e.g., concurrent: 1.32%; 95% CI: 1.21, 1.44%) and in stratified analyses (data not shown). Furthermore, we assessed whether associations differed when adjusted for individual exposure to PNC based on data from 290 visits with valid PNC measurements, but associations were similar overall and in stratified analyses, indicating no confounding by exposure to ultrafine particles (data not shown).

## Discussion

*Summary of results*. We investigated associations between 5-min averages of individual noise exposure from everyday life and HR and HRV. Associations differed for *L*_eq_ below and above 65 dB(A), but overall results support immediate effects of noise. HR and the LF:HF ratio were increased in association with concurrent noise exposure, with stronger associations for a 5-dB(A) increase in *L*_eq_ < 65 dB(A). SDNN increased in association with concurrent increases in noise < 65 dB(A) but decreased in association with lagged exposure, whereas noise ≥ 65 dB(A) was associated with concurrent reductions in SDNN only. LF and HF power decreased in association with concurrent noise < 65 dB(A), but decreased in association with concurrent noise and noise 0–5 min prior with increased levels of *L*_eq_ ≥ 65 dB(A). Associations also were modified by sex and age.

*Noise exposure and autonomic function*. The activity of the autonomic nervous system is reflected in HR and HRV, with higher levels of sympathetic input and lower levels of parasympathetic tone leading to increased HR and reduced HRV. The time-domain parameter SDNN reflects all periodic components of the variability of the HR. The contribution of sympathetic and parasympathetic activity can be separated, to some degree, by performing spectral analysis. It is generally accepted that HF power is mediated by the parasympathetic nervous system (Malik 1996), whereas the interpretation of LF power is controversial. In previous literature, LF power is often described solely as marker for sympathetic activity; however, LF power rather seems to be related to both the sympathetic and parasympathetic system (Stein and Kleiger 1999). Changes in the LF:HF ratio may provide information about the balance between sympathetic and parasympathetic modulations.

The observed immediate increase in HR and the LF:HF ratio associated with increases in *L*_eq_ < 65 dB(A) is consistent with parasympathetic withdrawal and/or elevated sympathetic tone, though the concurrent decrease in both HF and LF power is more consistent with a reduction in parasympathetic activity specifically. Subsequent increases in LF power after a delay of at least 10 min may indicate recovery of the autonomic nervous system. However, the immediate increase in SDNN followed by a decrease within 5 min is difficult to explain. In short-term recordings, not only LF and HF power but also very low frequency (VLF; *≤* 0.04 Hz) power can be determined as spectral components of HRV. The physiological correlates of VLF power are not well understood (Malik 1996). However, an additional analysis of the effect of noise exposure on VLF power indicated an immediate increase associated with a 5-dB(A) increase in noise < 65 dB(A) (14.6%; 95% CI: 12.6, 16.7%), but no delayed associations (data not shown). This suggests that the immediate increase in SDNN may have been the result of an increase in VLF power that was more pronounced than the concurrent decreases in LF and HF power. Furthermore, we speculate that this overreaction of the autonomic nervous system was regulated and returned to normal at least with a delay of 5 min.

Positive associations between HF power and increases in *L*_eq_ ≥ 65 dB(A) indicate an increase in parasympathetic activity. However, we also observed a small concurrent increase in HR that suggests an accompanying increase in sympathetic activity exceeding the parasympathetic modulation. Accordingly, the immediate elevation in the LF:HF ratio, which was only marginally significant, and the strong immediate increase in LF power also suggest increased sympathetic activity resulting in reduced HRV. This conclusion is further supported by the concurrent reduction in SDNN.

Because associations differed between low and high noise intensities, we assume different underlying mechanisms. Under participation of the limbic system and the hypothalamus, noise exposure is hypothesized to influence the autonomic nervous system either directly or indirectly through stress-induced hormone release (Babisch 2003; Babisch et al. 2001; Ising et al. 2003). As in the general noise-stress model (Henry 1992), a “fight-or-flight” response is activated by stressful situations, leading to the release of norepinephrine and other hormones that activate the synaptic transmission of sympathetic signals to the cardiac muscles fibers, in addition to increasing HR directly. Thus, changes in HRV associated with increases in lower noise intensities might be attributable mainly to parasympathetic withdrawal. In contrast, increases in higher noise intensities, which may be more stressful than comparable increases at lower levels of *L*_eq_, may lead to a transient reduction in HRV due to enhanced sympathetic activation and additional release of stress hormones. In the long run, any impairment in HRV may result in increased cardiovascular risk (Buccelletti et al. 2009; Gerritsen et al. 2001).

Previous studies of the effects of acute noise exposure on HRV are very limited and were mostly conducted in laboratory settings. Recently, Lee et al. (2010) exposed 16 healthy individuals to white noise of different intensities. In contrast with our findings, HR and HF power showed no changes in response to noise intensities ranging from background levels to 80 dB(A). LF power and the LF:HF ratio increased in response to white noise at 50 dB(A) or higher relative to mean values during exposure to background noise, and the LF:HF ratio tended to be higher during exposure to 70 and 80 dB(A) compared with 50 and 60 dB(A). Hence, at higher noise intensities increases in sympathetic activity may have been more pronounced than decreases in parasympathetic tone. Another laboratory study investigated the effects of white noise of 85 dB(A) on HRV in 20 young adults. The authors found an increase in total spectral power, a measure for total HRV, as well as an increase in LF power after 5–10 min of exposure, but no changes in HF power or HR (Björ et al. 2007). Nevertheless, laboratory studies do not reflect real-life conditions, which may explain the differences in results compared to epidemiological studies. A recent field study assessed the relation of night noise on respiratory sinus arrhythmia, which reflects HF power, as well as on pre-ejection period, a measure for sympathetic activity. The authors concluded that increased indoor traffic noise exposure levels during nighttime, which were < 30 dB(A), were associated with cardiac parasympathetic withdrawal, but not with changes in sympathetic tone (Graham et al. 2009). Even though these findings are consistent with our results, sleep is a state of reduced sympathetic activity and pronounced parasympathetic influence compared with waking hours, which complicates comparisons. Other epidemiological studies that estimated effects of short-term noise exposure on autonomic function reported positive associations with blood pressure and HR, suggesting an increase in sympathetic tone (Chang et al. 2003, 2009; Fogari et al. 2001; Haralabidis et al. 2008; Lusk et al. 2004). To our knowledge, only one previous study investigated possible effects of individual noise exposure during everyday life. Chang et al. (2009) conducted a study in 60 young adults who carried noise dosimeters and ambulatory blood pressure monitoring devices for 24 hr. A 5-dB(A) increase in environmental daytime noise with an average *L*_eq_ of 61.3 dB(A) was significantly associated with systolic (1.15 mmHG; 95% CI: 0.86, 1.43 mmHg) and diastolic (1.16 mmHg; 95% CI: 0.93, 1.38 mmHg) blood pressure (Chang et al. 2009).

*Stratified analyses*. Our study showed that sex significantly modified associations with increased noise < 65 dB(A), suggesting that women were more susceptible to increased noise exposure within the lower *L*_eq_ range than men. Because women were on average younger than men, these differences may have been confounded by age. However, stratified analyses by age group did not support this hypothesis. Existing studies on sex-specific effects have reported inconsistent results, with some reporting stronger associations in women (Bluhm et al. 2007; Chang et al. 2009; Heinonen-Guzejev et al. 2007; Willich et al. 2006), whereas others observed evidence of noise effects only in men (Babisch 2005; Barregard et al. 2009; Jarup et al. 2008), and at least two studies did not find sex differences at all (de Kluizenaar et al. 2007; Rosenlund et al. 2001). The inconsistencies may reflect differences among study populations, for example, regarding age and disease status, study designs, and measures of exposure.

Analyses stratified by age group suggested stronger effects of increases in noise < 65 dB(A) among those ≥ 65 years of age than in younger individuals. Hypertension was more common in the older age group, which may have increased susceptibility to effects of noise exposure on HRV. However, significant associations with increases in noise ≥ 65 dB(A) were only observed in those < 65 years.

*Strengths and limitations*. Participants had up to four repeated measurements with a mean duration of 6 hr. Calculating 5-min averages of *L*_eq_ and ECG parameters made a large number of repeated within-subject data available. By including a random effect for each person in the regression models, we were able to adjust for interindividual differences in ECG parameters and time-invariant characteristics such as sex and age. An additional strength is that we measured individual noise exposure, which may have substantially reduced exposure misclassification relative to previous studies that estimated noise exposure based on noise mapping. Another advantage of our study is that we performed a sensitivity analysis by additional adjustment for individually measured PNC. Traffic is a shared source of noise and air pollution and provides potential for confounding. Changes in HRV were already reported in association with PNC in diabetic participants of the same study population (Peters et al. 2010). However, adjusting for PNC had little or no influence on effect estimates for noise, consistent with previous studies (Beelen et al. 2009; Fuks et al. 2011). A further strength is that we examined high and low noise levels separately. The selection of 65 dB(A) as cut-off point was data driven. However, we consider the cut-off point as reasonable because the World Health Organization concluded that an average noise level of 65–70 dB(A) during the day is a possible threshold for a higher cardiovascular risk (Berglund et al. 1999).

Nevertheless, some limitations must be considered, including the potential for residual confounding. Depending on source and behavioral context, individuals may evaluate noise as annoying or even pleasant resulting in different physiological reactions. Nevertheless, we were not able to account for subjective annoyance because diary data were imprecise. A further limitation is that we were able to consider only PNC as potential confounder. Other pollutants were measured at a central monitoring site at a much lower time resolution. Therefore, they did not match our individual 5-min–based outcome data. Furthermore, ECG parameters strongly depend on movements and exercise of study participants. Even low-intensity exercise can increase HR and may produce higher noise intensities due to heavier respiration and rubbing of clothes. For this reason, it is essential to adjust for physical activity. Indeed, our variable reflecting physical activity was associated with all of the outcomes included in the analyses [see Supplemental Material, Table S7 (http://dx.doi.org/10.1289/ehp.1205606)]. However, our information on physical activity was based on self-report instead of actigraphy. Therefore, we adjusted for HR when estimating associations with HRV parameters. With regard to HR as response variable, it is not clear whether the adjustment for self-reported physical activity was sufficient; therefore, associations might be overestimated. Another limitation is that measurements of ECG parameters, noise, and PNC were temporally aligned based on the times recorded by each device and the study protocols. In case of inconsistencies in times, we confirmed with the study nurses and corrected the times wherever possible. Finally, the study population consisted mainly of elderly people and a lot of exclusions were made. Thus, generalizability to other populations might be restricted.

## Conclusions

Our study suggests acute changes in cardiac function in association with individual day-time noise exposure possibly mediated by a sympathovagal imbalance. Our results suggest that different biological pathways can be activated depending on noise intensity, and that noise at lower levels may have health consequences beyond those commonly attributed to “fight-or-flight” responses to high levels of noise.

## Correction

In the original manuscript published online, the values in Tables 3 and 4 had some rounding errors. They have been corrected here.

## Supplemental Material

(377 KB) PDF
