# Supplementary material for: Individual Daytime Noise Exposure during Routine Activities and Heart Rate Variability in Adults: A Repeated Measures Study
Source: Environ Health Perspect. 2013 Mar 19;121(5):607–12. doi: 10.1289/ehp.1205606 (PMC3672128; doi:10.1289/ehp.1205606)
Supplement: (377 KB) PDF [file ehp.1205606.s001.pdf]

Environmental Health Perspectives doi:10.1289/ehp.1205606

SUPPLEMENTAL MATERIAL

Individual Day-Time Noise Exposure During Routine Activities and Heart Rate Variability in Adults: A Repeated Measures Study

Ute Kraus, Alexandra Schneider, Susanne Breitner, Regina Hampel, Regina Rückerl, Mike Pitz,

Uta Geruschkat, Petra Belcredi, Katja Radon, Annette Peters

Table of contents:

	Page
**Supplemental Material:** Detailed description of the diary and physical activity.	2
**Supplemental Material, Table S1:** Final confounder models for each ECG parameter.	3
**Supplemental Material, Table S2:** Baseline characteristics of the study population by sex.	4
**Supplemental Material, Table S3:** Baseline characteristics of the study population by age-group.	5
**Supplemental Material, Table S4:** Description of diary entries	6
**Supplemental Material, Table S5:** Spearmen **c**orrelation coefficients for ECG parameters.	6
**Supplemental Material, Table S6:** Adjusted immediate and delayed associations between five-minute averages of L_{eq} and ECG measures.	7
**Supplemental Material, Table S7:** Associations of physical activity on HR as well as physical activity and HR on HRV parameters.	8
**Supplemental Material, Figure S1:** Number of observations and estimated exposure-response functions of immediate associations between five-minute averages of L_{eq} and ECG measures.	9

Detailed description of the diary and physical activity

During the measurement period between 7:30 a.m. and 3 p.m. participants were free to go whereever they liked and to pursue their daily routines. All their activities and whereabouts were recorded in a diary. In doing so, participants always made a diary entry when they changed their whereabouts or activity. Times were recorded precisely to the minute. A diary entry included a free text description of the activity. Furthermore, participants had to tick whether they were indoors, outside and not in traffic (e.g. in a park), or in traffic. Additionally, persons were asked to note when they felt annoyed by noise and to rate this annoyance on a scale with five levels ranging from "minor" to "extreme". After returning to the study center the nurses checked the diary for readability, completeness and conclusiveness. Every ambiguity was directly solved in discussion together with the participant. Dichotomous variables for the whereabouts where built.

To ensure that diary data can be aligned on the same timescale with exposure and outcome data, each participant got a wrist watch that was regularly synchronized with a radio controlled clock. The clocks of the exposure devices were likewise synchronized before starting the measurement. Furthermore, the study nurses recorded start and end times of the measurement periods in a protocol. Before combining the data times were compared with the times that were recorded by the study nurses.

TABLE S1. *Final confounder models for each ECG parameter*.

ECG parameter	Confounder model
HR	lagged HR, long-term time trend (linear), daily time trend based on every five minutes (smooth), physical activity (categorical)
SDNN	lagged SDNN, long-term time trend (polynomial, 2nd order), daily time trend based on every 30 minutes (polynomial, 4th order), physical activity (categorical), HR
LF power	lagged LF power, long-term time trend (linear), daily time trend based on every 15 minutes (polynomial, 4th order), physical activity (categorical), HR
HF power	lagged HF power, long-term time trend (linear), daily time trend based on every 15 minutes (polynomial, 4th order), physical activity (categorical), HR
LF/HF ratio	lagged LF/HF ratio, long-term time trend (polynomial 3rd order), daily time trend based on every 5 minutes (smooth), physical activity (categorical), HR

Abbreviations: HF, high frequency; HR, heart rate; LF, low frequency; SDNN, standard deviation of normal-to-normal intervals

TABLE S2. *Baseline characteristics of the study population by sex.*

Characteristic	Men		Women		*P*
	N	(% or mean ± SD)	N	(% or mean ± SD)	
Age [yrs]	69	(63.7 ± 11.1)	41	(58.1 ± 11.9)	0.016[e]
Body mass index [kg/m^2]	69	(28.8 ± 4.7)	41	(28.2 ± 6.4)	0.59[e]
Smoking history					
Never smoker	45	(65.2)	14	(34.1)	0.0016[f]
Ex smoker	24	(34.8)	27	(65.9)	
Metabolic disorder (T2D[a] or IGT[a])	42	(60.9)	22	(53.7)	0.46[f]
Self-reported history[b]					
Myocardial infarction	6	(8.7)	0	(0.0)	0.08[g]
Angina pectoris	2	(2.9)	4	(9.8)	0.19[g]
Coronary heart disease	6	(8.7)	1	(2.4)	0.25[g]
Hypertension	42	(60.9)	19	(46.3)	0.14[f]
Use of medication[c]					
Agents acting on renin-angiotensin-system	27	(39.1)	13	(31.7)	0.43[f]
Beta blocker	21	(30.4)	7	(17.1)	0.12[f]
Calcium channel blockers	8	(11.6)	3	(7.32)	0.53[g]
Antidiabetics	14	(20.3)	4	(9.8)	0.15[f]
Diuretics	24	(34.8)	12	(29.3)	0.55[f]
Nitrates	1	(1.5)	0	(0.0)	1.00[g]
Statins	16	(23.2)	3	(7.3)	0.033[f]
Antihypertensive drugs	38	(55.1)	16	(39.0)	0.10[f]
Hearing impairment[d] (%)	12	(17.4)	3	(7.3)	0.14[f]
If yes: Physician diagnosed	9	(13.0)	3	(7.3)	1.00[g]
Wearing hearing aid	2	(2.9)	0	(7.3)	1.00[g]
Employed (%)	24	(34.8)	17	(41.5)	0.48[f]

[a]Participants with T2D were classified based on self-report of a diagnosis by a physician, self-reported medication use, or a fasting glucose level >125mg/dl or 2h glucose level ≥200mg/dl in an oral glucose tolerance test (OGTT). IGT was specified as having 2h OGTT glucose levels ≥140mg/dl but <200mg/dl.
[b]Ever physician diagnosed.
[c]At least once during the study period (Mar 17th 2007 to Dec 17th 2008).
[d]Not validated.
P-values determined with [e]Student's *t*-test, [f]chi-square test or [g]Fisher's exact test.
Abbreviations: T2D, type 2 diabetes; IGT, impaired glucose tolerance; SD, standard deviation.

TABLE S3. *Baseline characteristics of the study population by age-group.*

Characteristic	< 65 years		≥ 65 years		*P*
	N	(% or mean ± SD)	N	(% or mean ± SD)	
Age [yrs]	55	(52.1 ± 8.6)	55	(58.1 ± 11.9)	<.0001[d]
Body mass index [kg/m^2]	55	(28.3 ± 6.3)	55	(28.9 ± 4.3)	0.55[d]
Men	29	(52.7)	40	(72.7)	0.030[e]
Smoking history					
Never smoker	28	(50.9)	23	(41.8)	
Ex smoker	27	(49.1)	32	(58.2)	0.34[e]
Metabolic disorder (T2D[a] or IGT[a])	23	(41.8)	41	(74.6)	0.0005[f]
Self-reported history[b]					
Myocardial infarction	1	(1.8)	5	(9.1)	0.21[f]
Angina pectoris	4	(7.3)	2	(3.6)	0.68[f]
Coronary heart disease	4	(7.3)	3	(5.5)	1.00[f]
Hypertension	23	(41.8)	38	(69.1)	0.0040[e]
Use of medication[c]					
Agents acting on renin-angiotensin-system	14	(25.5)	26	(47.3)	0.017[e]
Beta blocker	7	(12.7)	21	(38.2)	0.0022[e]
Calcium channel blockers	3	(5.5)	8	(15.6)	0.11[e]
Antidiabetics	7	(12.7)	11	(20.0)	0.30[e]
Diuretics	12	(21.8)	24	(43.6)	0.015[e]
Nitrates	0	(0.0)	1	(1.8)	1.00[f]
Statins	4	(7.3)	15	(27.3)	0.0055[e]
Antihypertensive drugs	18	(32.7)	36	(65.5)	0.0006[e]
Hearing impairment[d] (%)	1	(1.8)	14	(25.5)	0.0003[e]
If yes: Physician diagnosed	1	(100.0)	11	(78.6)	1.00[f]
Wearing hearing aid	0	(0.0)	2	(14.3)	1.00[f]
Employed (%)	38	(69.09)	3	(5.5)	<.0001[e]

[a]Participants with T2D were classified based on self-report of a diagnosis by a physician, self-reported medication use, or a fasting glucose level >125mg/dl or 2h glucose level ≥200mg/dl in an oral glucose tolerance test (OGTT). IGT was specified as having 2h OGTT glucose levels ≥140mg/dl but <200mg/dl.
[b]Ever physician diagnosed.
[c]At least once during the study period (Mar 17th 2007 to Dec 17th 2008).
[d]Not validated.
P-values determined with [e]Student's *t*-test, or [f]chi-square test.
Abbreviations: T2D, type 2 diabetes; IGT, impaired glucose tolerance; SD, standard deviation.

TABLE S4. *Description of diary entries (N=4,148).*

Diary based information	Diary entries N (%)	5-minute segments N (%)
Whereabouts		
Indoors	2,268 (54.78)	14,020 (65.5)
Outside, not in traffic	159 (3.8)	917 (4.3)
In traffic	1,687 (40.7)	4,904 (22.9)
Unclear	34 (0.8)	1,578 (7.4)
Physical activity		
Sleeping/Reclining	110 (2.7)	329 (1.5)
Very light/light exertion	3,766 (90.8)	20,032 (93.5)
Moderate/vigorous/heavy exertion	272 (6.6)	1,058 (4.9)

TABLE S5. *Spearmen correlation coefficients for ECG parameters.*

ECG measures	HR	SDNN	LF	HF	LF/HF Ratio
HR	1	-0.22	-0.18	-0.31	0.15
SDNN		1	-0.21	-0.16	-0.02
LF			1	0.41	0.40
HF				1	-0.59
LF/HF Ratio					1

Abbreviations: HF, high frequency; HR, heart rate; LF, low frequency; SDNN, standard deviation of normal-to-normal intervals.

TABLE S6. *Adjusted immediate and delayed associations between five-minute averages of L_{eq} and ECG measures.*

ECG measures	< 65 dB(A) % change (95%CI)	≥ 65 dB(A) % change (95%CI)
HR		
concurrent	1.48 (1.37, 1.60)*	0.18 (0.05, 0.31)*
0-5min	0.29 (0.17, 0.41)*	0.09 (-0.04, 0.22)
5-10min	0.12 (0.01, 0.24)*	0.08 (-0.04, 0.21)
10-15min	0.09 (-0.02, 0.21)	0.15 (0.02, 0.28)*
SDNN		
concurrent	5.74 (5.13, 6.36)*	-0.67 (-1.30, -0.04)*
0-5min	-0.53 (-1.12, 0.05)	-0.08 (-0.71, 0.56)
5-10min	-0.69 (-1.26, -0.12)*	-0.09 (-0.73, 0.54)
10-15min	-0.67 (-1.26, -0.13)*	-0.21 (-0.84, 0.43)
LF power		
concurrent	-3.77 (-5.49, -2.02)*	4.42 (2.59, 6.32)*
0-5min	0.26 (-1.53, 2.09)	3.69 (1.86, 5.56)*
5-10min	2.14 (0.37, 3.95)*	1.50 (-0.30, 3.33)
10-15min	2.24 (0.49, 4.02)*	1.74 (-0.07, 3.57)
HF power		
concurrent	-8.56 (-10.31, -6.78)*	2.89 (0.95, 4.87)*
0-5min	-1.31 (-3.21, 0.62)	3.45 (1.50, 5.44)*
5-10min	0.87 (-1.01, 2.79)	1.58 (-0.34, 3.55)
10-15min	1.90 (-0.04, 3.80)	1.67 (-0.26, 3.63)
LF/HF ratio		
concurrent	4.89 (3.48, 6.32)*	1.38 (0.03, 2.75)*
0-5min	0.98 (-0.38, 2.36)	-0.09 (-1.43, 1.26)
5-10min	0.96 (-0.36, 2.31)	-0.18 (-1.52, 1.17)
10-15min	0.12 (-1.17, 1.43)	0.05 (-1.29, 1.40)

**P*-value of fixed effect for L_{eq} as piecewise linear term in additive mixed model < 0.05

Abbreviations: dB(A), A-weighted decibels; change, change of outcome mean per 5 dB(A) increase in noise exposure; CI, confidence interval; HR, heart rate; SDNN, standard deviation of normal-to-normal intervals; HF, high frequency; LF, low frequency; min, minute

Table S7. *Associations of physical activity on HR as well as physical activity and HR on HRV parameters.*

outcome	exposure	% change[a]	(95% CI)
HR	moderate PA	1.67*	(0.67, 2.67)
	high PA	6.49*	(5.38, 7.59)
SDNN	HR	-0.35*	(-2.03, -1.42)
	moderate PA	-10.50*	(-54.22, -27.93)
	high PA	-15.85*	(-67.56, -45.09)
LF power	HR	-3.92*	(-18.83, -17.39)
	moderate PA	12.31	(-13.54, 269.41)
	high PA	28.36*	(55.03, 682.96)
HF power	HR	-4.22*	(-20.13, -18.62)
	moderate PA	-1.48	(-57.25, 101.58)
	high PA	8.36	(-37.08, 254.73)
LF/HF ratio	HR	0.34*	(1.02, 2.35)
	moderate PA	11.24	(-1.33, 194.06)
	high PA	15.43*	(11.43, 276.75)

[a]%-change in outcome mean per increase in physical activity category compared to the lowest activity level and per increase of 1 beat/min in HR, respectively.

*p-value<0.05

Abbreviations: CI, confidence interval; HR, heart rate; SDNN, standard deviation of normal-to-normal intervals; HF, high frequency; LF, low frequency; PA, physical activity

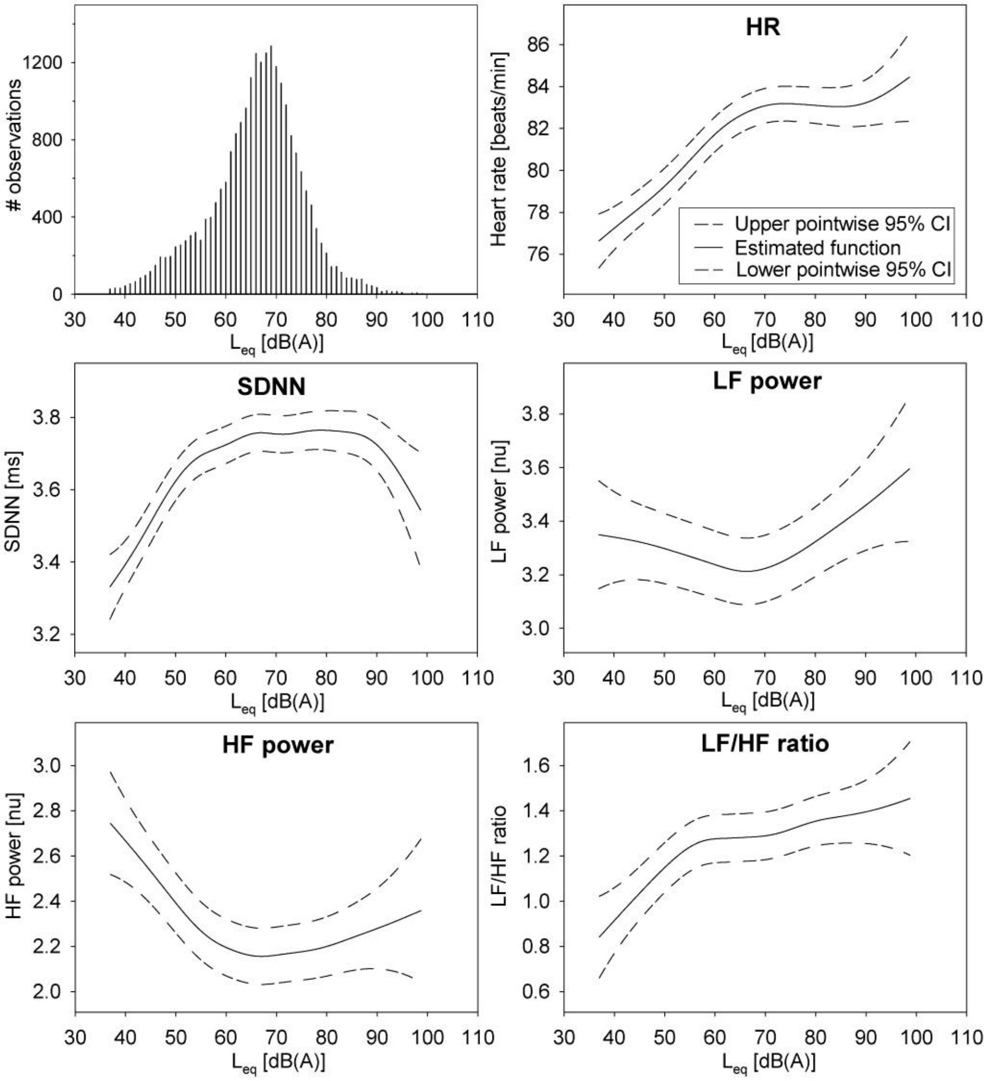

FIGURE S1. *Number of observations (upper left panel) and estimated exposure-response functions of immediate associations between five-minute averages of L_{eq} and ECG measures.* Abbreviations: CI, confidence interval; dB(A), A-weighted decibels; HF, high frequency; HR, heart rate; L_{eq}, equivalent continuous sound pressure levels; LF, low frequency; nu, normalized units; SDNN, standard deviation of normal-to-normal intervals.
